# Nutritional Value of Savory Herb (*Satureja hortensis* L.) and Plant Response to Variable Mineral Nutrition Conditions in Various Phases of Development

**DOI:** 10.3390/plants9060706

**Published:** 2020-06-01

**Authors:** Natalia Skubij, Katarzyna Dzida, Zbigniew Jarosz, Karolina Pitura, Monika Jaroszuk-Sierocińska

**Affiliations:** 1 Institute of Horticulture Production, Subdepartment of Plant Nutrition, University of Life Sciences in Lublin, 28 Głęboka Street, 20-612 Lublin, Poland; natalia.skubij@gardendc.pl (N.S.); zbigniew.jarosz@up.lublin.pl (Z.J.); karolina.pitura@up.lublin.pl (K.P.); 2 Institute of Soil Science, Environment Engineering and Management, University of Life Sciences in Lublin, 7 Leszczyńskiego Street, 20-069 Lublin, Poland; monika.jaroszuk@up.lublin.pl

**Keywords:** macroelements, ionic ratios, nitrogen dose, mineral status, abiotic stress

## Abstract

Mineral nutrition and plant ontogeny influence both the physiological balance between nutrients in a plant and determine the proper nutritional status of a plant, which is necessary to realize the yielding potential of a cultivated species. The aim of the present study was to assess the effect of nitrogen doses (0, 4, 8, 12, 16 g N·m^−2^) and plant development phases (the beginning and full flowering) on the content of macroelements and changes in ionic ratios occurring in the herb of the summer savory cv. ‘Saturn’. The two-factor experiment was carried out in a random-block design with five replications. The mineral nitrogen nutrition applied increased the concentration of total nitrogen and its mineral forms in the plants. There was a change in ion homeostasis in the individual stages of the ontogenesis process, i.e., a higher content of P, K, Ca, and S in the initial flowering phase as well as Mg and Cl in the full flowering phase. The increase in the availability of mineral nitrogen in the soil solution caused a decrease in total sorption capacity, reducing the potential of the soil for saturation with alkaline cations.

## 1. Introduction

In the current millennium, the improvement of human health is a priority for many individuals dealing with food, including herb, vegetable, and fruit growers. Mineral elements play an important role in growth, as they help to maintain fitness and prevent human diseases [1]. Among crops, herbs are valuable sources of easily absorbable mineral substances [2]. Summer savory (*Satureja hortensis* L.) is a widely known annual herbaceous plant from the Lamiaceae family cultivated in many countries worldwide. The herb of this plant has carminative and digestive properties as well as antidiarrheal, antispasmodic, and antibacterial activity [3]. Due to the healing effects and a significant nutritional value, the herbaceous parts of the stems and leaves of this species are used in the food, medical, and pharmaceutical industries [1,4]. The composition of *Satureja hortensis* L. raw material has been found to contain significant amounts of minerals, including potassium (1.68–3.38 mg∙kg^−1^ DM), phosphorus (0.31–0.72 mg∙kg^−1^ DM), calcium (1.08–2.84 mg∙kg^−1^ DM), magnesium (0.25–0.61 mg∙kg^−1^ DM), iron (242–726 mg∙kg^−1^ DM), and sodium (0.007–0.013 mg∙kg^−1^ DM) [5,6,7,8,9]. Given the high amount of mineral elements in the aerial parts of *S. hortensis* L. [2], it can potentially serve as a dietary source of human-essential minerals.

The mineral status of plants is important not only for the nutritional value of food, but also for the growth, development, and yielding of crops [1]. Plants are exposed to the influence of adverse environmental factors, which are known as abiotic stresses, excessive soil salinity, and the absence of mineral salts. Mineral nutrition is one of the environmental factors causing changes in the ionic balance in the sorption complex and in the soil solution, but also affecting the assimilation process and concentration of nutrients in plant tissues. At the same time, too intensive mineral fertilization is considered one of the causes of anthropogenic soil salinity, which causes salt stress in plants [10]. The salts of carbonic, sulfuric, and hydrochloric acids as well as calcium, magnesium, and sodium bases are responsible for changing the degree of saturation of the soil solution with mineral components [11]. The direct interaction of the salt causes a decrease in water availability, lowering its potential in soil solution [12]. As a result, plants growth is limited most often as a result of plant ionic balance disturbances or osmotic stress [13]. The excess amounts of salts accumulated in the chloroplasts can exert toxic effects on photosynthesis through destabilization of the protein complex and the destruction of photosynthetic pigments, leading to oxidative stress in plants [10,14]. However, both the excess and deficiency of specific mineral nutrient induce a number of disorders in the metabolism of plants, preventing the use of its full crop-forming potential. An insufficient content or absence of nutrients available to plants contribute to disorders of plasma membrane stability, which leads to e.g., changes in their selective permeability to ions and organic compounds produced by the process of photosynthesis [13].

Mineral nutrition may induce the development of abiotic stress in plants; on the other hand, it helps to neutralize the effects of stress. An optimal amount of nutrients supports the proper functioning of plants, which allows their organisms to increase tolerance through the regeneration of damage or starting alternative metabolic pathways. Moreover, nitrogen fertilization has a significant impact on plant resistance, as it indirectly participates in the production of secondary metabolites in support of defense response to stress. However, it directly affects the synthesis of proteins, including specific protein classes such as proteins affecting membrane properties, calcium binding proteins, osmoprotectant accumulation proteins, chaperones, and defense proteins type late embryogenesis abundant (LEA) [10]. The task of these proteins is to participate in mechanisms that increase plant resistance. In addition, nitrogen is one of the basic elements forming the plant structure. Most (75%) of this nutrient is contained in leaves, mainly in chloroplasts, as part of ribulose-1,5-bisphosphate carboxylase (RuBisCO) [15]. It plays an important role in the production of a substantial amount of plant biomass; additionally, it is a component of amino acids, proteins, nucleic acids, coenzymes, amides, chlorophyll, and cytokinins [16,17]. It participates in the transport of protons and electrons in photosynthesis and respiration and in the path of signal transduction between organs [17]. Nitrogen balance in plants also defines the quantitative demand for minerals necessary to maximize the efficiency of a given physiological process [18]. The importance of nitrogen in plant development was confirmed by Mumivand et al. [7], who used increasing doses of nitrogen and calcium and noted an increase in the concentration of nitrogen, phosphorus, calcium, magnesium, iron, manganese, and zinc in the herb of *Satureja hortensis* L. cv. Saturn. Szewczuk and Mazur [19] studied the effect of nitrogen and developmental phase on the content of mineral components in nettle leaves and stems. They found a significant effect of the increasing doses of nitrogen (up to 300 kg N∙ha^−1^) on the content of nitrates and manganese in the plants in all development phases. It has also been shown that nitrogen fertilization not only increases the N content in plant tissues, but also changes the concentration of other nutrients [20,21,22,23].

The change in the accumulation of nutrients in the plant is somewhat due to the different rates of uptake by the roots from the soil solution [24,25]. These differences cause that cations and anions accumulate in plants in uneven amounts, demonstrating synergistic or antagonistic interactions [26]. The individual functions of particular mineral elements are important for the proper course of biochemical changes and physiological reactions during the plant development cycle [25,27]. In addition to the direct effects of ingredients on plant metabolism, their interaction is also important and often more important than their individual action [26,28]. Thus, the mineral nutrient uptake is also influenced by ontogenetic variability, which is not limited to the variability of plant development phases, but occurs within the phases as well [29]. The accumulation of mineral compounds and biomass gain do not proceed simultaneously but have a species-specific rhythm and dynamics. Ontogenetic diversity is especially important, as it largely determines the time of harvest and the chemical composition and the health-enhancing properties of raw material [8,19].

In times of climate change and the intense increase in the food needs of the growing population, it is important to develop agricultural practices that will increase plant resistance to changing environmental conditions. Considering various determinants of the mineral composition of plants, detailed research was carried out into the impact of varied nitrogen fertilization and phase of summer savory development on the content of macroelements in the raw material. The main interactions between individual mineral components in the savory raw material inducing a change in its mineral state were analyzed as well. Soil sorption properties determining the uptake of minerals by plants from the soil solution were also investigated.

## 2. Results and Discussion

### 2.1. Nitrogen in Savory Raw Material

**Figure 1:**
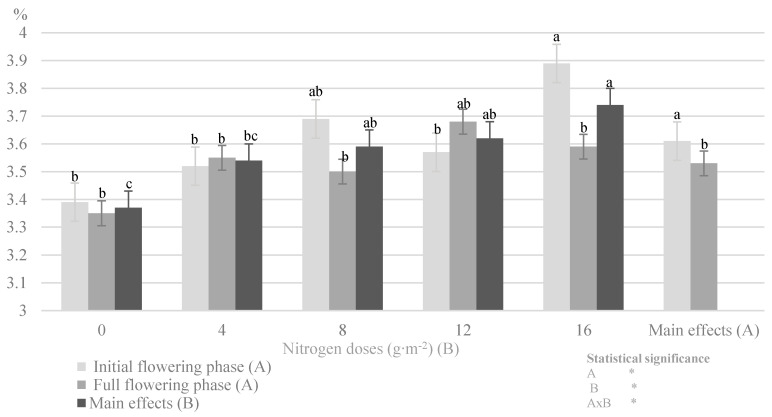
Impact of the nitrogen dose and plant development phase on total nitrogen content in the herb savory cv. Saturn (in 2014–2016) (Note: The results are presented as the mean ± SD; means sharing the same letter on a bars do not differ significantly according to Tukey’s test at *p* ≤ 0.05; significant effects for the main factors and for interaction between them are indicated with asterisks (*); n.s.—no significance).

Among the factors of plant growth and development, nitrogen is included in the group of the main (superior) and supporting (detailed) factors [18] that affect the metabolic processes in a plant by modifying the amount of biomass produced in a given development phase [17]. In the present experiment, there was a significant effect of the nitrogen dose and the plant development phase on total nitrogen content (from 3.35% to 3.89% DM) (Figure 1) and its two mineral forms (NH_4_^+^ and NO_3_^−^) (Table 1) in the herb of the summer savory cv. Saturn. A positive effect of nitrogen fertilization on nitrogen accumulation was found in the summer savory [7,30], basil [31], coriander, Italian fennel, and star anise [32], sagebrush tarragon [9], caraway [21], sand plantain [33], and rosemary [34].

The herbs in the initial flowering phase were characterized by a higher total nitrogen concentration. Golcz et al. [35] reported a similar relationship in their research on different cultivars of basil, i.e., increased content of nitrogen in the initial flowering phase relative to full flowering. A significant impact of the development cycle on the change in the nitrogen concentration was found in *Thymus vulgaris* L., *Melisa officinalis* L. [36], and in *Urtica dioica* L. [19]. The variation in the nitrogen value during the development cycle of *Satureja hortensis* L. was associated with nitrogen remobilization in the plant during the growing season. The soil is the main source of N uptake during the vegetative phase, whereas nitrogen assimilated in chloroplasts is utilized at the time of development of generative organs [18]. Therefore, a decrease in the content of total nitrogen was noted in the subsequent stages of development of the summer savory. This relationship was also related to the availability of nitrogen forms. Regardless of the type of mineral fertilizer, plants absorb nitrogen as a cation (NH_4_^+^) or an anion (NO_3_^−^) [16,24]. Nitrates are the preferred nitrogen sources for most crop plants. However, before nitrate is included in the metabolic processes of the synthesis of organic nitrogen compounds, it must be reduced with the participation of nitrate reductase. Under the influence of increasing doses of nitrogen, this enzyme, shows a variable intensity of action in individual plant organs, with the strongest activity in stems and leaf blades at the early stages of vegetation [24]. In the present experiment, the savory cv. Saturn plants in the initial flowering phase were characterized by higher nitrate nitrogen levels. Szewczuk and Mazur [19] reported a similar relationship between the developmental stages of the common nettle, in which the vegetative and flower bud stages exhibited higher levels of NO_3_^−^ anions than the flowering phase.

Along with the increase in the nitrogen dose, the content of both studied forms of nitrogen increased in the Saturn cv. herb (Table 1). An increase in the concentration of the analyzed forms of nitrogen after the application of differential nitrogen fertilization was noted in the leaves of basil cv. Kasia and cv. Wala [31], garden savory herb [30], and rocket leaves [37,38]. Plant nitrogen uptake preferences depend on the species and environmental factors [23,39]. An important issue is the synergistic and antagonistic relationship between the forms of nitrogen and the cations or anions taken up by plants [26,40].

### 2.2. Other Macroelements Present in the Herb

The nitrogen functions in the plant also involve determination of the physiological balance between nitrogen and other minerals [28,40]. Macronutrients regulate many metabolic processes and indirectly influence nitrogen metabolism; hence, they play an important role in the crop-forming potential of cultivated plants [18]. The analysis of the nutrient content in the aboveground parts of the summer savory cv. Saturn showed a number of significant relationships (Table 2).

**Figure 2:**
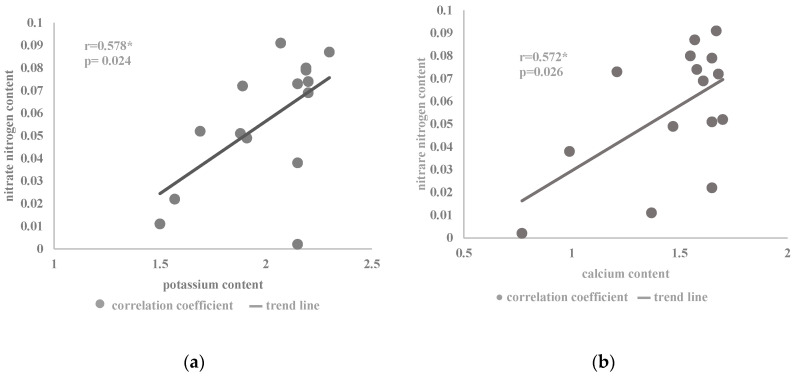
Coefficient of correlation between the content of nitrate nitrogen (% DM) and the content of (**a**) potassium (**b**) calcium in the herb of the summer savory cv. Saturn in relation to the nitrogen dose and plant development phases (Table 2) (* correlation at *p* < 0.05).

The content of potassium in the savory herb was on average 2.01% DM and was significantly dependent on the applied nitrogen dose. An increase in the concentration of potassium ions was recorded in plants fed with the lower doses of nitrogen. A similar tendency of the influence of nitrogen on the assimilation of potassium cations was noted in the savory [7,30] and sand plantain [33]. An inverse relationship was found for *Ocimum basilicum* L. cv. Kasia and cv. Wala [31] as well as for *Rosmarinus officinalis* L. [34], in which plants fertilized with higher doses of nitrogen were characterized by a higher concentration of potassium. In the conducted tests, an increased amount of potassium was noted in plants harvested at the initial stage of flowering. A similar result of a higher amount of potassium in the earlier stages of development than at the time of full flowering was reported by Karimi et al. [8], who analyzed the aboveground and underground parts of raw summer savory. Research conducted by Szewczuk and Mazur [19] showed that the raw material of nettle harvested in the vegetative and flower bud stages was characterized by higher K content than in the flowering phase. The variable potassium accumulation in the savory cv. Saturn plants after applying higher doses of nitrogen is also associated with ionic interactions between the nutrients [17,24]. Potassium is usually present in the plant in quite a high concentration, especially in meristematic tissues and in the phloem. As a nutrient, it participates in osmoregulation and affects, among others, the regulation of the activity of enzymes determining the nitrogen balance in plants [41]. Changing the availability of potassium in a plant organism causes disturbances in nitrogen metabolism, inducing a change in the proportion between nitrogen fractions [16]. Potassium has a positive interaction with the NH_4_^+^ form; however, it is also an accompanying cation during the transport of the NO_3_^−^ anion in the xylem from the root system, where it accumulates to the aboveground parts [16]. In the summer savory cells, a positive correlation between K^+^ and NO_3_^−^ was noted (Figure 2a). In field conditions, the interaction of nitrogen and potassium is associated with an increase in the efficiency of nitrogen fertilizers [42]. The relationship between nitrogen and potassium ions was also confirmed by Chen et al. [25] and Rietra et al. [26]. Magnesium uptake intensity also induced a change in the assimilation of potassium ions in savory plants. These nutrients show ionic competition for a protein carrier and an ion channel in transport across the cell membrane [17,28]. As a result, there are noticeable disturbances in the uptake of both these elements (Table 1) during the application of increasing doses of nitrogen in the initial flowering phase.

**Figure 3:**
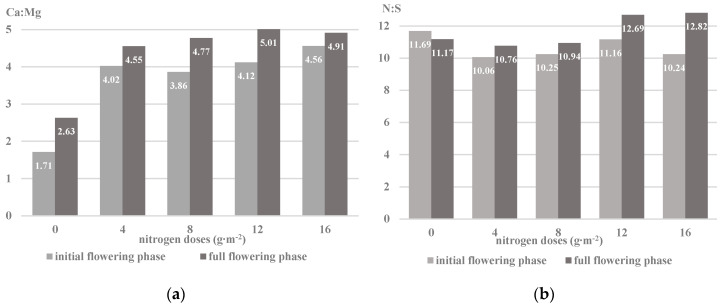
Ionic interaction of nutrients in the herb of the savory of Saturn cv. in the plant development phases depending on the dose of nitrogen: (**a**) ionic ratio between Ca and Mg (**b**) ionic ratio between N total and S (based on the mean for 2014–2016).

In both development phases of the Saturn cv. plants, a decrease in the magnesium content and an increase in the calcium concentration under the influence of the increasing nitrogen doses were recorded. An important determinant of the assimilation of the ions of these elements is their antagonistic interaction. An optimal relationship between these nutrients is noted at the Ca:Mg ratio in the range from 5:1 to 8:1 [18,28]; at a wider ratio, the Mg^2+^ uptake is reduced. Thus, the increase in the concentration of nitrogen in the soil solution had an impact on the regulation of magnesium and calcium uptake in the savory plant cells (Figure 3a). An antagonistic interaction of calcium and magnesium ions has been reported in studies by Soltani et al. [9] in plants of *Artemisia dracunculus* L. A variable assimilation of Ca^2+^ and Mg^2+^ relative to different doses of nitrogen was also observed in *Satureja hortensis* L. cv. Saturn [7] and *Ocimum basilicum* L. [43]. At the same time, the analysis of the impact of the development cycle in the studied cultivar revealed a greater concentration of magnesium and calcium ions in the initial flowering phase (Table 2). A similar relationship was noted in nettle raw material, which was characterized by a higher content of calcium and magnesium in the vegetative phase [19]. However, Karimi et al. [8] reported a greater number of ions of the nutrients tested in *Satureja hortensis* L. herb harvested before flowering than during the flowering phase. 

In the present experiment, a positive correlation was found between the content of calcium cations and nitrate nitrogen anions in the *Satureja hortensis* L. ‘Saturn’ herb under the influence of the analyzed factors (Figure 2b). The synergistic interaction in the assimilation of calcium ions and nitrates, also presented in the literature [17,26], is associated with an increase in calcium uptake by plants when the amount of nitrates relative to ammonium ions predominates in the soil solution. This relationship results from the inhibitory influence of NH_4_^+^ ions on Ca^2+^ absorption. It may also result from the fact that nitrate nutrition increases the production of organic acids and, indirectly, the uptake of cations [40]. The cations neutralize the negative charge transfer effect of OH^−^ groups on non-volatile organic acids to maintain cytoplasmic acidity. This reaction is the result of a nitrate reduction process prior to their incorporation into plant metabolism [40]. This process is also important, as it increases the uptake of other cations such as potassium, which was also noted in the ionic correlation with nitrates in the savory herb (Figure 2a).

As a component of cell membranes, coenzymes, and buffers, phosphorus participates in photosynthesis and in the metabolism of proteins, carbohydrates, and fats [44]. In addition, it plays an important role in energy storage and maintenance of the structural integrity of membranes [25]. In the herb of the summer savory cv. ‘Saturn’, an increase in the assimilation of phosphorus ions was recorded after application of the increasing doses of nitrogen in relation to the control plants (Table 2). A similar relationship was found in the leaves of *Zingiber officinale* Rosc. [29]. In turn, a decrease in the phosphorus uptake induced by nitrogen treatment was described by Jalili [22] in summer savory and by David and Rothstein [45] in Fraser fir. A higher phosphorus value was obtained in the initial flowering phase of these plants versus the full flowering phase. The action of phosphorus proceeds throughout the vegetation period, but it is particularly pronounced during the phase of development of the plant root system and the plant maturation stages [46]. During the aging of plants, phosphorus is remobilized from older leaves to younger ones or the developing organs, e.g., seeds [18]. 

**Figure 4:**
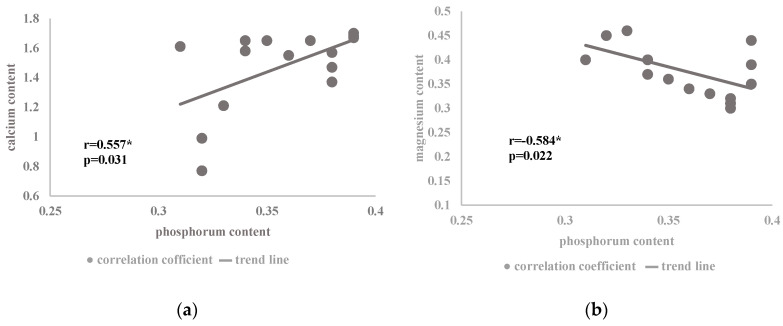
Coefficient of correlation between the content of phosphorus (% DM) and the content of (**a**) calcium and (**b**) magnesium (% DM) present in the mineral composition of the herb of the summer savory cv. Saturn in relation to the nitrogen dose and plant development phases (Table 2) (* correlation at *p* < 0.05).

A positive correlation between phosphorus and calcium (Figure 4a) and a negative correlation between phosphorus and magnesium (Figure 4b) were found in the studied *Satureja hortensis* L. species influenced by the analyzed factors. The relationship between calcium and phosphorus is associated with the fact that mineral phosphorus forms can occur in soil in an exchangeable form and in compounds with calcium, iron, or aluminum. However, the soil reaction is a decisive factor for the use of the particular forms of phosphorus by plants. At the same time, calcium phosphates dissolve more easily in an acidic environment, where their optimum solubility is in the range of pH 5–6 [47]. Since ammonium nitrate fertilizers used in the study can change soil acidity (Table 3), the applied increasing dose of nitrogen contributed to the creation of an environment conducive to the solubility of calcium phosphate forms, hence the possible increase in the content of calcium and phosphorus ions in plant cells. The analysis of the soil used for growing the savory cv. Saturn in the individual developmental phases showed a significant effect of the nitrogen dose on changes in the concentration of mineral components in the soil solution (Table 4). A similar relationship in the assimilation of calcium and phosphorus ions was noted for *Cucumis sativus* L. cv. Al-Hytham [48]. On the other hand, the interactions between P and Ca are related to the fact that calcium is a cofactor of phospholipases involved in protein phosphorylation [18].

However, the relationship between the increase in the phosphorus content and the decrease in magnesium assimilation in the savory plants (Figure 4b) is associated with the synergistic interaction of phosphorus ions with calcium (Figure 4a). On the other hand, calcium exhibits antagonistic interactions with magnesium ions, which was evident in the present study (Figure 3a).

Sulfur is a structural component of sulfur amino acids, determining the formation of the secondary and tertiary structure of proteins affecting the activity of the nitrogenase and nitrate reductase enzymes [49]. This element influences the course of photosynthesis by regulating the amount of ferredoxin, i.e., a biological electron transmitter, which can lead to reduction in the intensity of nitrate (V) degradation and the accumulation of mineral forms of nitrogen in the plant [50]. The sulfur content in the herb of the savory cv. Saturn ranged from 0.28% to 0.38% DM, depending on the factors examined. The plants accumulated more sulfur in the initial flowering phase than those harvested in the full flowering phase. A similar tendency of sulfur assimilation in the raw material collected in the initial flowering phase was noted for *Melissa officinalis* L., *Salvia officinalis* L., and *Thymus vulgaris* L. after applying NPK mineral fertilization in a two-year vegetation cycle [36]. In the present experiment, there was a visible dependence of the increase in the sulfur concentration in the plants under the influence of the increased nitrogen dose in relation to the control objects. The assimilation of sulfate ions was affected by the value of the N:S ion ratio, which regulates the plant sulfur requirements in relation to its nitrogen demand (the optimal N:S ratio is 10:1 to 12:1) (Figure 3b). The narrowing of the N:S ratio in the plant leads to the accumulation of inorganic sulfur compounds, while the expansion of the N:S ratio increases the uptake of non-protein nitrogen forms. Thus, the optimal level of sulfur allows reduction of the share of non-protein forms of nitrogen in the plant by converting simple forms of nitrogen into proteins [50]. The dependence of ionic sulfur assimilation on nitrogen was also reported in other plant species: *Brassica campestris* L. cv. Pusa Gold, *Eruca sativa* Mill. [51], *Brassica napus* L. [52], and *Zea mays* L. [53]. 

In the savory Saturn cv. plants, an increase in the chlorine content was recorded under the influence of the increasing nitrogen doses (Table 2). Both the nitrogen form and chlorine ions affect the ionic balance in the plant. The monovalent nitrate and chloride anions balance out; therefore, the more chlorides, the less nitrates [18,38]. Therefore, with the increase in nitrate anion assimilation, a decrease in the uptake of chloride ions was noted in the herb Saturn cv.; the most intense interaction was visible for the dose of 4 g N∙m^−2^ (Table 1 and Table 2). This antagonism between Cl^−^ and NO_3_ ions was confirmed in the research by Kafkafi et al. [44] on tomato plants and Bar et al. [54] on avocado, lemon, and mandarin trees. At the same time, the chlorine anion plays an important role in nitrogen metabolism, in which it is responsible for the activation of aspartic synthetase and protein synthesis [55], as well as in the Hill reaction and ATP production during photosynthesis. In addition, this element is an osmoregulator taking part in the opening and closing of stomata along with potassium [27]. Due to its functions, the main site of chlorine accumulation is the vegetative parts of plants. Therefore, the conducted experiment showed a greater assimilation of chloride anions in the *Satureja hortensis* L. plants in the initial flowering phase than in the full flowering phase. This is confirmed by the results of Kafkafi et al. [41], in which vegetative organs (leaves, stems, root) were characterized by higher chlorine content than generative parts (fruit, seeds) of mature cotton plants. 

### 2.3. Nutrients Available in Soil

Appropriate proportions of cations and anions in the soil sorption complex are one of the basic conditions for the proper nutrition of plants. Violation of these proportions may adversely affect the yield and chemical composition of herbal raw material [18,24]. The analysis of soil from the cultivation of the summer savory cv. Saturn showed significant differences in the nutrient content (Table 4). 

Higher concentrations of ammonium nitrogen, phosphorus, potassium, calcium, magnesium, sulfur, and chlorine ions were found in the soil from plants harvested at full flowering. The nitrate nitrogen content was higher in the initial flowering phase. In a study carried out by Nurzyńska-Wierdak and Dzida [56], a higher content of the above-mentioned cations and anions was found in the substrate from the cultivation of marjoram in the flower bud phase. The fertilization of plants with nitrogen had a significant impact on the change in the uptake of components from the soil solution. In the present experiment, the increase in the concentration of chlorides, sulfates, and phosphates was associated with the increasing dose of nitrogen. Nitrates were an exception, as their content in the soil solution decreased due to the increased mineral fertilization. The increase in the concentration of nitrates in plants is affected by significant accumulation of sulfates in the substrate, resulting from their impaired ability to reduce and further processing [50]. However, an increase in the concentration of chloride ions in the soil solution increases the activity of cations, contributing to an increase in their uptake [27]. In the soil from the summer savory cultivation, there was an increased absorption of calcium and magnesium cations, while the activity of potassium and ammonium nitrogen uptake in relation to the applied nitrogen doses was lower. The roots of dicotyledons, such as the *Satureja hortensis* L. cv. ‘Saturn’, have a higher sorption capacity, taking more divalent than monovalent cations [47], hence the possible increase in calcium and magnesium ion accumulation by the plants studied. At the same time, the sum of exchangeable cations (SEC) in the soil decreased relative to the analyzed research factors (Table 3). The content of exchangeable cations showed certain regularity in the soil solution (Table 4); on this basis, they can be ranked as follows: Ca^2+^ > K^+^ > Mg^2 +^ > NH_4_^+^. In contrast, hydrolytic acidity (HA) referring to the amount of acid cations present in soil was dependent on the applied nitrogen dose. On average, the highest values were obtained for the highest dose in both studied stages of development. The increase in the mineral fertilization caused a decrease in the total sorption capacity (TSC), reducing the soil potential for the adsorption of cations during the vegetative process of the summer savory plants.

**Figure 5:**
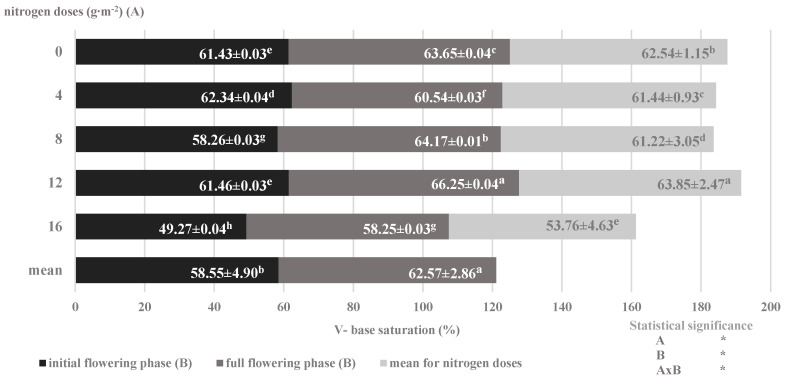
Degree of saturation of soil with basic cations depending on nitrogen nutrition and development phases of savory plants (mean for 2014–2016) (Note: The results are presented as the mean ± SD; Means sharing the same letter on a bars do not differ significantly according to Tukey’s test at *p* ≤ 0.05; significant effects for the main factors and for interaction between them are indicated with asterisks (*); n.s.—no significance).

At the same time, there was a decrease in the saturation of the sorption complex with cations relative to the increasing nitrogen dose, with a simultaneous increase in their accumulation, which was greater in the full flowering phase than in the initial flowering phase (Figure 5). The most common explanation of this phenomenon in the literature is the physiologically acidic effect of ammonium nitrate as well as the desorption and leaching of basic cations [47]. The place of base cations (Ca^2 +^, Mg^2 +^, K^+^) is occupied by hydrogen ions (acid cations) secreted to the soil by plant roots, which, according to the perverse rule, leads to desorption of cations from the soil sorption complex [18]. Thus, the decrease in the soil sorption capacity and changes in the degree of saturation with basic cations, as well as the share of individual cations in the sorption complex and therefore in the soil solution, were differentiated by the conditions of plant nutrition and the accumulation of individual ions in plants during the ontogenesis process.

The salt concentration (EC) in the soil environment and appropriate proportions between individual nutrients are extremely important for the proper uptake of water and ingredients dissolved in soil [31,34]. The highest EC value was found after applying the highest nitrogen dose in the initial flowering phase (Table 3). A similar relationship was found in soil used for growing other plant species, e.g., *Thymus vulgaris* L. and *Satureja hortensis* L. [57] as well as *Ocimum basilicum* L. [31]. The comparison of the mineral composition in the plants and in the soil on which they were grown to the concentration of salts in the substrate demonstrated that the anion and cation uptake by the savory plants was not reduced at the increasing value of EC. As reported by Esmaili et al. [58], an excessive salt concentration in the substrate results in reduction in the N, P, Ca, and Mg uptake and an increased concentration of chlorine in plant tissues. However, differences in nutrient uptake by plants also arise under the influence of other factors, such as the soil type or weather conditions. It has also been found that nitrogen fertilization increases plant tolerance to excessive salinity through an improvement in plant nutritional status [58]. The results obtained in this study indicate that nitrogen fertilization facilitated proper nutrient uptake and absorption by the summer savory plants, also during the increasing concentration of salts in the growing soil.

Among many factors affecting the growth and yielding of cultivated plants, soil acidity is mentioned as one of the most important determinants. The optimal pH range for most crops is from 5.5 to 6.5 [18]. In the present experiment, the soil reaction (pH) after harvesting the summer savory cv. Saturn was within the optimal range and increased under the influence of the increasing dose of nitrogen (Table 3). An inverse relationship in the effects of nitrogen on the pH of the soil solution was found in *Ocimum basilicum* L. [31] as well as *Thymus vulgaris* L. and *Satureja hortensis* L. [57].

It is commonly known that the soil reaction has a major influence on the amount of organic matter (C_org_). An increase in pH leads to an increase in organic matter solubility, while soil acidification can have either a positive or negative effect. However, in this research, the content of organic matter of soil depends on the balance between many environmental processes and factors, which may be the cause of the low dependence between C_org_ and pH.

## 3. Material and Methods

### 3.1. Plant Material and Experimental Conditions

The field experiments with the summer savory cv. Saturn were carried out in 2014–2016 in an agricultural farm in Zamch (50°18′ N, 23°1′ E), i.e., a village located in southeastern Poland, Lublin Province. The research area is characterized by the shortest the early winter period in the Lublin Province (below 32 days; vegetation period 220–250 days), the highest average air temperatures, and the high water evaporation values. During the years of experimentation, the site had a temperature range from 7.6 to 21.3 °C and a rainfall range from 12 to 135 mm∙month^−1^. The data on the atmospheric conditions in 2014–2016 were provided by the meteorological station at the RSVE in Nowy Lubliniec.

The summer savory plants were grown on Stagnic Luvisols developed from silt formations. The soil was classified in the good wheat complex and bonitation class IIIb. The granulometric composition of the soil was silt loam (the fraction content according to BN-78/9180-11). The chemical composition of the soil before sowing (mg∙dm^−3^) was as follows: 22 P-PO_4,_ 46.15 K, 249.5 Ca, 38.85 Mg, 18.75 S-SO_4_, pH 6.29, and also EC 0.072 (mS∙cm^−1^).

In all years, the experiment was established in a two-factor random-block design in five replications. The area of a single microplot was 1 m^2^. Each plot was separated by 0.5 m walkways. The direct sowing experiment was established with the amount of 1 g of seeds per m^2^. The seeds of the cultivar were produced in the Institute of Natural Fibers and Medicinal Plants on Poznań. The seed sowing date was dependent on the weather conditions determining the onset of the vegetation period (21.05.2014, 10.05.2015, 03.05.2016).

The first study factor was the dose of nitrogen, i.e., 0, 4, 8, 12, and 16 g N·m^−2^, while the second variable was the term of the raw material harvest, i.e., the initial flowering phase and the full flowering of plants. Plant fertilization was supplemented with a dose of magnesium (4 g Mg·m^−2^), potassium (10 g K·m^−2^), and phosphorus (3 g P·m^−2^). The first mineral fertilization treatment was applied a week before sowing the seeds with the following nitrogen first doses: 2N, 4N, 6N, and 8N g·m^−2^. Additionally, each plot was fertilized with 3 g P·m^−2^, 5 g K·m^−2^, and 2 g Mg·m^−2^. About 21 days after seedling emergence, a second fertilization treatment was applied. It consisted of a second dose of nitrogen (2, 4, 6, and 8 g N·m^−2^) and the remaining dose of potassium and magnesium. No additional P+K+Mg fertilization was applied in plots with the dose of 0 g N·m^−2^, as these served as the control objects. The mineral following mineral fertilizers were used in the experiment: ammonium nitrate (34% N), potassium salt (49.8% K), triple granulated superphosphate (20% P), and magnesium heptahydrate sulfate (9.6% Mg; 12.8% S).

During the experiment, the plants were manually weeded without using any herbicides to fight the weed infestation. During the growing season, no diseases or pests were found; therefore, no protection measures were applied.

The first harvest term was established in the first 10 days of August 2014, the second 10 days of August 2015, and the third 10 days of July 2016, i.e., at the beginning of plant flowering. The second harvest was carried out in the full flowering phase, i.e., at day 14 after the onset of flowering (20 August 2014, 1 September 2015, and 4 August 2016).

### 3.2. Plant Analysis

Plant material samples were collected immediately after plant harvest; the material was dried at a temperature of 60 °C and ground prior to the determinations of the mineral composition of the plants. The total N was determined with the Kjeldahl method, after burning the material in H_2_SO_4_ in a Kjeltec System 2002 Distilling Unit automatic analyzer. The contents of N-NH_4_ and N-NO_3_ were determined in dry matter (DM) after using the Bremners distillation method, modified by Starck. To determine P, K, Ca, and Mg, the plant material was burnt dry at a temperature of 550 °C. After cooling, the ash was acidified with hydrochloric acid diluted at a ratio of 1:2, and the content of the above-mentioned nutrients was determined. The content of K, Ca, and Mg was determined using atomic absorption spectrometry (ASA) (Aanalyst 300, Perkin Elmer, ‎Waltham, MA, USA). Phosphorus was determined colorimetrically with ammonium metavanadate. Moreover, the content of S-SO_4_ and Cl was analyzed colorimetrically in a 2.0% acetic acid extract: sulfates with barium chloride and chlorine with silver nitrate. Based on the average results for the individual mineral components, ionic ratios between calcium and magnesium and between nitrogen and sulfur were calculated.

### 3.3. Soil Analysis

After harvest, the collected soil samples were analyzed in 0.03 M CH_3_COOH to determine N-NH_4_, N-NO_3_, phosphorus, potassium, calcium, magnesium, sulfur, and chlorine using the same methods as in the plant material. The soil samples were taken from Ap horizons (from the depth of 0–10 cm). In the cultivated soil, we determined: reaction (pH), potentiometrically, hydrolytic acidity (HA), and sum of exchangeable cations (SEC) using the Kappen method. On its basis, we calculated the value of soil total sorption capacity (TSC), organic carbon (Corg) with the Ťiurin method with modification proposed by Simakov, and the salt concentration in the cultivated soil (EC) with the conductometric method.

### 3.4. Statistical Analysis

Two-way analysis of variance (ANOVA) was performed to analyze the effects of the nitrogen doses and development phases on savory herbs. The least significant difference was determined for mean comparison at a 5% level of significance (t-Tukey test). The potential interactions between mineral nutrients were analyzed using Pearson’s correlation coefficient (*p* ≤ 0.05) (Statistica 13.1 Software). The data are expressed as the mean ± standard deviation (SD). All data were analyzed using the Statistica (Analytical Software, 13.1., PL).

## 4. Conclusions

In conclusion, the mineral nutrition significantly modified the concentration of nutrients in the herb of *Satureja hortensis* L. cv. Saturn. The highest concentration of most of the analyzed macroelements was noted after application of the lower nitrogen doses (4–8 g N·m^−2^) in the savory raw material harvested at the initial stage of flowering. At the same time, the developmental changes occurring during ontogenesis had an effect on the intensity of nutrient accumulation and determined the ionic relationships between mineral components in the Saturn cv. plants. An influence of soil sorption properties on the anion and cation uptake from the soil solution depending on the factors studied was noted as well. The study contributed to the determination of the optimal limits of nitrogen nutrition and the development phase that is appropriate for harvesting the raw material of the garden savory cv. Saturn; hence, the results are applicable in agricultural practice. Concurrently, the research factors used have an impact on the quality of the savory raw material and allow determination of the proper nutritional value of the herb, which is increasingly being used as a supplement to the diet in the rapidly growing human population.

## Figures and Tables

**Table 1:** Effect of the nitrogen dose and plant development phases on the content of nitrogen forms of summer savory herb (in 2014–2016) (Note: The results are presented as the mean ± SD; means sharing the same letter in a column do not differ significantly according to Tukey’s test at *p* ≤ 0.05; significant effects for the main factors and for interaction between them are indicated with asterisks (*); n.s.—no significance).

Nitrogen Dose (g∙m^−2^) (B)	N-NH_4_ (% DM)			N-NO_3_ (% DM)		
	Plant Development Phases (A)					
	Beginning of Flowering	Full Flowering	Main Effects (B)	Beginning of Flowering	Full Flowering	Main Effects (B)
0	0.054 ± 0.031 ^bc^	0.013 ± 0.001 ^cd^	0.028 ± 0.034 ^b^	0.073 ± 0.039 ^ab^	0.002 ± 0.001 ^c^	0.038 ± 0.045 ^bc^
4	0.045 ± 0.026 ^bc^	0.065 ± 0.036 ^ab^	0.055 ± 0.032 ^a^	0.080 ± 0.009 ^ab^	0.069 ± 0.010 ^ab^	0.074 ± 0.010 ^a^
8	0.021 ± 0.006 ^c^	0.043 ± 0.020 ^b^	0.032 ± 0.018 ^b^	0.091 ± 0.042 ^a^	0.052 ± 0.008 ^b^	0.072 ± 0.035 ^ab^
12	0.092 ± 0.002 ^a^	0.020 ± 0.001 ^c^	0.056 ± 0.037 ^a^	0.079 ± 0.017 ^ab^	0.022 ± 0.003 ^c^	0.051 ± 0.032 ^b^
16	0.015 ± 0.006 ^c^	0.039 ± 0.002 ^bc^	0.027 ± 0.013 ^b^	0.087 ± 0.021 ^a^	0.011 ± 0.002 ^c^	0.049 ± 0.042 ^b^
**Main effects (A)**	0.045 ± 0.033 ^a^	0.034 ± 0.028 ^b^		0.082 ± 0.027 ^a^	0.031 ± 0.026 ^b^	
Statistical significance						
A			*			*
B			*			*
AxB			*			*

**Table 2:** Effect of the nitrogen dose and plant development phases on the chemical composition of summer savory herb (in 2014–2016) (Note: For an explanation, see Table 1).

Plant Development Phases (A)	Nitrogen Dose (g∙m^−2^) (B)	P	K	Ca	Mg	S	Cl
		(% DM)					
Beginning of flowering	0	0.33 ± 0.029 ^bc^	2.15 ± 0.066 ^b^	1.21 ± 0.22 ^d^	0.46 ± 0.055 ^a^	0.29 ± 0.014 ^bc^	0.57 ± 0.116
	4	0.36 ± 0.036 ^b^	2.19 ± 0.056 ^a^	1.55 ± 0.02 ^cd^	0.34 ± 0.013 ^c^	0.35 ± 0.012 ^ab^	0.34 ± 0.079
	8	0.39 ± 0.042 ^a^	2.07 ± 0.117 ^b^	1.67 ± 0.11 ^ab^	0.35 ± 0.013 ^c^	0.36 ± 0.017 ^ab^	0.45 ± 0.050
	12	0.37 ± 0.024 ^ab^	2.19 ± 0.061 ^a^	1.65 ± 0.19 ^b^	0.33 ± 0.036 ^c^	0.32 ± 0.026 ^b^	0.73 ± 0.218
	16	0.38 ± 0.030 ^ab^	2.30 ± 0.026 ^a^	1.57 ± 0.07 ^c^	0.32 ± 0.010 ^c^	0.38 ± 0.041 ^a^	0.85 ± 0.288
**Full flowering**	0	0.32 ± 0.063 ^bc^	2.15 ± 0.039 ^b^	0.77 ± 0.07 ^e^	0.45 ± 0.030 ^a^	0.30 ± 0.022 ^bc^	0.58 ± 0.125
	4	0.31 ± 0.041 ^c^	2.20 ± 0.086 ^a^	1.61 ± 0.29 ^bc^	0.40 ± 0.014 ^b^	0.33 ± 0.012 ^b^	0.35 ± 0.056
	8	0.39 ± 0.024 ^a^	1.69 ± 0.052 ^c^	1.70 ± 0.32 ^a^	0.44 ± 0.021 ^a^	0.32 ± 0.025 ^b^	0.57 ± 0.103
	12	0.34 ± 0.012 ^bc^	1.57 ± 0.106 ^c^	1.65 ± 0.38 ^b^	0.40 ± 0.014 ^b^	0.29 ± 0.007 ^c^	0.69 ± 0.284
	16	0.38 ± 0.026 ^ab^	1.50 ± 0.074 ^c^	1.37 ± 0.22 ^d^	0.30 ± 0.011 ^c^	0.28 ± 0.010 ^c^	0.80 ± 0.291
**Main effects** Beginning of flowering Full flowering		0.37 ± 0.031 ^a^	2.18 ± 0.318 ^a^	1.53 ± 0.21	0.36 ± 0.061 ^b^	0.34 ± 0.039 ^a^	0.59 ± 0.251
		0.35 ± 0.040 ^b^	1.82 ± 0.309 ^b^	1.42 ± 0.43	0.40 ± 0.057 ^a^	0.30 ± 0.025 ^b^	0.60 ± 0.242
**Nitrogen dose**							
**0**		0.32 ± 0.047 ^b^	2.15 ± 0.052 ^a^	0.99 ± 0.28 ^b^	0.45 ± 0.043 ^a^	0.30 ± 0.018 ^b^	0.57 ± 0.117 ^b^
**4**		0.34 ± 0.045 ^ab^	2.20 ± 0.069^a^	1.58 ± 0.20 ^a^	0.37 ± 0.033 ^b^	0.34 ± 0.016 ^a^	0.34 ± 0.067 ^c^
**8**		0.39 ± 0.030 ^a^	1.89 ± 0.214 ^b^	1.68 ± 0.23 ^a^	0.39 ± 0.055 ^b^	0.35 ± 0.030 ^a^	0.51 ± 0.101 ^b^
**12**		0.35 ± 0.021 ^ab^	1.88 ± 0.335 ^b^	1.65 ± 0.29 ^a^	0.36 ± 0.046 ^b^	0.30 ± 0.025 ^b^	0.71 ± 0.248 ^a^
**16**		0.38 ± 0.026 ^a^	1.91 ± 0.424 ^b^	1.47 ± 0.18 ^a^	0.31 ± 0.014 ^c^	0.33 ± 0.061 ^a^	0.82 ± 0.283 ^a^
Statistical significance							
A		*	*	n.s.	*	*	n.s.
B		*	*	*	*	*	*
AxB		*	*	*	*	*	n.s.

**Table 3:** Cation exchange properties as well as pH and EC in the soil after the summer savory harvest (mean for 2014–2016) (Note: For an explanation, see Table 1).

Plant Development Phases (A)	Nitrogen Dose (g∙m^−2^) (B)	HA	SEC	TSC	EC	C_org_	pH_H_2_O_
		cmol(+)·kg^−1^			(mS∙cm^−1^)	(%)	
**Beginning of flowering**	0	3.16 ± 0.04 ^ab^	5.11 ± 0.04 ^b^	8.27 ± 0.02 c	0.087 ± 0.008	0.772 ± 0.004 ^d^	5.85
	4	3.15 ± 0.04 ^b^	5.33 ± 0.04 ^a^	8.48 ± 0.04 ^a^	0.115 ± 0.001	0.817 ± 0.002 ^c^	5.90
	8	2.78 ± 0.03 ^c^	3.91 ± 0.02 ^f^	6.69 ± 0.03 ^f^	0.126 ± 0.033	0.777 ± 0.002 ^d^	5.81
	12	3.21 ± 0.02 ^ab^	5.16 ± 0.04 ^b^	8.37 ± 0.04 ^b^	0.121 ± 0.029	0.842 ± 0.001 ^b^	5.97
	16	3.22 ± 0.02 ^a^	3.12 ± 0.03 ^g^	6.34 ± 0.03 ^g^	0.145 ± 0.034	0.893 ± 0.003 ^a^	5.89
**Full flowering**	0	2.78 ± 0.05 ^c^	4.90 ± 0.03 ^c^	7.78 ± 0.03 ^d^	0.085 ± 0.014	0.603 ± 0.003 ^h^	6.03
	4	2.80 ± 0.02 ^c^	4.32 ± 0.02 ^e^	7.12 ± 0.03 ^e^	0.101 ± 0.011	0.617 ± 0.003 ^g^	6.20
	8	2.43 ± 0.04 ^d^	4.31 ± 0.02 ^e^	6.74 ± 0.03 ^f^	0.108 ± 0.020	0.847 ± 0.002 ^b^	6.33
	12	2.42 ± 0.03 ^d^	4.72 ± 0.03 ^d^	7.14 ± 0.04 ^e^	0.130 ± 0.046	0.662 ± 0.002 ^f^	6.33
	16	2.81 ± 0.02 ^c^	3.91 ± 0.02 ^f^	6.72 ± 0.04 ^f^	0.121 ± 0.029	0.763 ± 0.002 ^e^	6.19
**Main effects** Beginning of flowering Full flowering		3.10 ± 0.17 ^a^	4.53 ± 0.87 ^a^	7.63 ± 0.93 ^a^	0.119 ± 0.034	0.820 ± 0.045 ^a^	5.81–5.97
		2.65 ± 0.18 ^b^	4.44 ± 0.35 ^b^	7.08 ± 0.37 ^b^	0.109 ± 0.029	0.699 ± 0.094 ^b^	6.03–6.33
Nitrogen dose							
0		2.97 ± 0.20 ^a^	5.01 ± 0.11 ^a^	7.98 ± 0.28 ^a^	0.086 ± 0.011 ^b^	0.689 ± 0.089 ^e^	5.85–6.03
4		2.98 ± 0.18 ^a^	4.83 ± 0.52 ^c^	7.80 ± 0.71 ^b^	0.108 ± 0.025 ^ab^	0.716 ± 0.107 ^d^	5.90–6.20
8		2.60 ± 0.17 ^c^	4.11 ± 0.20 ^d^	6.72 ± 0.39 ^d^	0.117 ± 0.028 ^a^	0.811 ± 0.046 ^b^	5.81–6.33
12		2.81 ± 0.41 ^b^	4.94 ± 0.22 ^b^	7.75 ± 0.61 ^c^	0.126 ± 0.032 ^a^	0.752 ± 0.096 ^c^	5.97–6.33
16		3.01 ± 0.21 ^a^	3.52 ± 0.41 ^e^	6.53 ± 0.20 ^e^	0.133 ± 0.036 ^a^	0.828 ± 0.069 ^a^	5.89–6.19
**Statistical significance**							-
**A**		*	*	*	n.s	*	
**B**		*	*	*	*	*	
**AxB**		*	*	*	n.s	*	

**Table 4:** Nutrient content in the soil after the summer savory harvest (in 2014–2016) (Note: For an explanation, see Table 1).

Plant Development Phases (A)	Nitrogen Dose (g∙m^−2^) (B)	N-NH_4_	N-NO_3_	P-PO_4_	K	Ca	Mg	S-SO_4_	Cl
		(mg∙dm^−3^)							
**Beginning of flowering**	0	10.75 ± 0.54 ^cd^	36.57 ± 0.62 ^ab^	22.64 ± 1.08 ^d^	50.65 ± 0.51	331.83 ± 1.53	35.85 ± 0.44	19.02 ± 0.65 ^c^	46.96 ± 0.93 ^b^
	4	15.95 ± 0.38 ^bc^	27.65 ± 0.39 ^bc^	31.08 ± 0.89 ^bc^	65.75 ± 0.69	330.50 ± 2.09	50.57 ± 0.62	22.12 ± 0.74 ^c^	26.46 ± 0.86 ^c^
	8	11.90 ± 0.98 ^cd^	54.07 ± 0.67 ^a^	31.57 ± 0.94 ^bc^	67.95 ± 1.03	306.00 ± 1.49	48.77 ± 0.37	21.60 ± 0.62 ^c^	46.10 ± 0.43 ^b^
	12	14.52 ± 0.75 ^bc^	24.50 ± 0.48 ^bc^	35.63 ± 0.84 ^ab^	65.45 ± 0.79	317.00 ± 1.98	42.30 ± 0.26	36.80 ± 0.88 ^ab^	63.63 ± 0.56 ^a^
	16	16.45 ± 0.40 ^b^	25.20 ± 0.51 ^bc^	30.13 ± 1.05 ^bc^	58.80 ± 0.80	297.16 ± 0.92	53.35 ± 0.83	34.45 ± 0.64 ^ab^	42.24 ± 0.37 ^b^
**Full flowering**	0	013.47 ± 0.94 ^bcd^	10.75 ± 0.54 ^c^	33.24 ± 0.52 ^ab^	63.82 ± 0.52	365.66 ± 1.48	47.00 ± 0.19	23.50 ± 0.82 ^bc^	21.55 ± 0.33 ^d^
	4	48.75 ± 0.66 ^d^	14.35 ± 0.48 ^c^	34.77 ± 0.72 ^ab^	83.45 ± 072	373.16 ± 1.86	60.90 ± 0.10	33.05 ± 0.53 ^ab^	31.87 ± 0.28 ^c^
	8	815.72 ± 0.41 ^bc^	13.65 ± 0.36 ^c^	33.88 ± 0.35 ^ab^	70.90 ± 0.88	431.66 ± 2.12	59.87 ± 0.49	48.90 ± 0.73 ^a^	64.35 ± 0.42 ^a^
	12	1217.85 ± 1.03 ^ab^	22.05 ± 0.45 ^bc^	37.34 ± 0.83 ^a^	83.25 ± 0.90	335.16 ± 1.70	55.00 ± 0.34	32.95 ± 0.43 ^bc^	69.31 ± 1.18 ^a^
	16	1621.87 ± 0.76 ^a^	24.15 ± 0.50 ^bc^	29.67 ± 0.89 ^c^	70.87 ± 0.35	367.16 ± 1.62	60.62 ± 0.39	31.87 ± 0.59 ^bc^	51.06 ± 0.80 ^b^
**Main effects** Beginning of flowering Full flowering Nitrogen dose 0 4 8 12 16		13.91 ± 0.61^b^	33.60 ± 1.02 ^a^	30.21 ± 0.95 ^b^	61.72 ± 0.84 ^b^	316.50 ± 1.64 ^b^	46.17 ± 0.96 ^b^	26.80 ± 0.98 ^a^	45.08 ± 0.62
		15.53 ± 1.13 ^a^	16.99 ± 0.66 ^b^	33.78 ± 0.89 ^a^	74.46 ± 0.97 ^a^	374.56 ± 1.97 ^a^	56.68 ± 1.16 ^a^	34.05 ± 1.02 ^a^	47.63 ± 0.73
		12.11 ± 0.94 ^b^	23.66 ± 0.41 ^ab^	27.94 ± 1.06 ^c^	57.23 ± 0.78	348.75 ± 1.40	41.42 ± 0.67 ^c^	21.26 ± 0.35 ^b^	34.25 ± 0.78 ^d^
		12.33 ± 0.28 ^b^	21.00 ± 0.30 ^b^	32.92 ± 0.72 ^b^	74.60 ± 0.59	351.83 ± 1.03	55.73 ± 0.69 ^a^	27.58 ± 0.31 ^ab^	29.16 ± 0.38 ^d^
		13.81 ± 0.59 ^b^	33.86 ± 0.84 ^a^	32.73 ± 0.69 ^b^	69.42 ± 0.43	368.83 ± 1.66	54.32 ± 0.72 ^ab^	35.25 ± 0.73 ^a^	55.23 ± 0.20 ^b^
		16.18 ± 0.60 ^ab^	23.27 ± 0.45 ^ab^	36.49 ± 0.95 ^a^	74.35 ± 0.47	326.08 ± 1.25	48.65 ± 0.73 ^b^	34.87 ± 0.39 ^a^	66.47 ± 0.54 ^a^
		19.16 ± 0.81 ^a^	24.67 ± 0.21 ^ab^	29.90 ± 0.99 ^bc^	64.83 ± 0.34	332.17 ± 0.92	56.98 ± 0.81 ^a^	33.16 ± 0.55 ^a^	46.65 ± 0.59 ^c^
Statistical significance									
A		*	*	*	*	*	*	*	n.s.
B		*	*	*	n.s.	n.s.	*	*	*
AxB		*	*	*	n.s.	n.s.	n.s.	*	*
